# Emergency Psychiatric Effects of Nicotine Vaping Cessation: A Narrative Review

**DOI:** 10.7759/cureus.103116

**Published:** 2026-02-06

**Authors:** John Wahidy, Alexander Mazzorana, Laith Fada, Greg Jacobs

**Affiliations:** 1 Medicine, Alabama College of Osteopathic Medicine, Dothan, USA; 2 Emergency Medicine, Alabama College of Osteopathic Medicine, Dothan, USA

**Keywords:** acute psychiatric, electronic cigarettes, emergency psychiatry, nicotine replacement therapy, nicotine withdrawal, substance related psychiatric disorder

## Abstract

Nicotine vaping represents a common form of nicotine use among adolescents and adults. Nicotine dependence associated with vaping can develop rapidly. Emergency departments (EDs) are increasingly encountering patients with acute anxiety, mood instability, agitation, and other psychiatric symptoms. Abrupt cessation of nicotine vaping may be an under-recognized contributor in these cases due to withdrawal.

This narrative aims to synthesize evidence on acute psychiatric effects following nicotine vaping cessation and nicotine withdrawal, with a focus on emergency and other acute-care settings. This objective builds on the growing recognition of nicotine withdrawal as a contributor to acute psychiatric presentations.

This descriptive review summarizes PubMed-indexed English-language literature published from January 2010 to December 2025, including systematic reviews, observational studies, and clinical trials relevant to nicotine withdrawal treatment. It also covers ED and intensive care unit (ICU) studies where nicotine withdrawal presents as agitation or delirium, as well as case reports describing severe psychiatric presentations connected to e-cigarette use or withdrawal.

Across study types, nicotine withdrawal is consistently associated with anxiety, irritability or agitation, depressed mood or anhedonia, sleep disturbance, and difficulty concentrating. In acute care environments, nicotine withdrawal has also been associated with agitation or delirium phenotypes in hospitalized and ICU populations. Furthermore, e-cigarette use has been linked to increased odds of suicidality and psychotic-like symptoms in some observational studies. Finally, case literature documents severe psychotic presentations that are temporally associated with e-cigarette use or nicotine withdrawal, although causality is difficult to establish.

Nicotine vaping cessation can plausibly precipitate acute psychiatric symptoms that mimic primary psychiatric illness and complicate ED triage and disposition. As a result, consistent nicotine-use history-taking, withdrawal-aware differential diagnosis, and judicious use of nicotine replacement therapy (NRT) may increase diagnostic reliability and patient safety.

## Introduction and background

Electronic nicotine delivery systems (ENDS), including e-cigarettes, are now a dominant nicotine source for many youths and a substantial minority of adults. US surveillance has repeatedly shown a high prevalence of youth vaping and frequent use patterns [1-3]. Dependence is not limited to combustible cigarettes. Validated instruments establish clinically meaningful dependence symptoms among adolescent and young adult e-cigarette users. These include craving and difficulty quitting [4-6]. Nicotine withdrawal is defined in the Diagnostic and Statistical Manual of Mental Disorders, Fifth Edition (DSM-5) and International Classification of Diseases (ICD) frameworks by a characteristic symptom cluster, including irritability, restlessness, insomnia, anxiety, and impaired concentration following cessation or reduction of nicotine use, regardless of delivery form.

From an emergency psychiatry standpoint, nicotine withdrawal is a diagnostic "shape shifter." It can present as panic-like anxiety, dysphoria, agitation, insomnia, irritability, or impaired concentration. These symptoms overlap with primary mood or anxiety disorders, stimulant intoxication or withdrawal, akathisia, emerging psychosis, or delirium. Withdrawal symptoms can also amplify baseline psychiatric vulnerabilities. For example, high anxiety sensitivity or low distress tolerance can create a high-risk window for impulsive behavior and suicidal ideation in certain patients [7,8].

This narrative review focuses on the acute psychiatric consequences of nicotine vaping cessation, emphasizing ED and acute care relevance. In the absence of extensive vaping-specific emergency data, conclusions are anchored in the broader nicotine withdrawal literature to ensure relevance and rigor.

## Review

Methods

This narrative review covers PubMed-indexed literature from January 2010 to December 2025. Searches were conducted using combinations of the terms nicotine withdrawal, vaping cessation, electronic cigarettes, ENDS, psychiatric symptoms, emergency department, agitation, delirium, suicidal ideation, and psychosis. The review was undertaken to address the limited emergency department-specific literature on psychiatric outcomes following vaping cessation. We prioritized several areas: (1) epidemiology of vaping and dependence; (2) ENDS pharmacology and dependence mechanisms; (3) studies on nicotine withdrawal symptom profiles and trajectories; (4) acute-care and ICU literature describing agitation, delirium, and withdrawal management; (5) systematic reviews or meta-analyses linking e-cigarette use to suicidality and psychosis-related outcomes; and (6) vaping cessation intervention trials relevant to ED bridging care. As this is a narrative synthesis rather than a systematic review, we did not conduct a formal risk-of-bias assessment, and we explicitly address limitations. Findings are synthesized narratively under thematic headings relevant to emergency and acute-care psychiatry.

Neurobiology and pathophysiology linking vaping cessation to acute psychiatric symptoms

This section was informed by literature identified using the terms nicotine withdrawal, vaping cessation, ENDS, nicotine dependence, and neurobiology.

Nicotine is highly reinforcing and can rapidly drive dependence by stimulating reward pathways in the brain. Its effects on mood, attention, and arousal contribute to clinically significant withdrawal when use is abruptly discontinued [9]. Contemporary ENDS can deliver nicotine efficiently. In some designs, they can approach or exceed the nicotine delivery of traditional cigarettes, increasing nicotine dependence [9-11]. Nicotine delivery varies substantially, with measures in cartridges ranging from less than 1 mg to nearly 20 mg, and aerosolized nicotine yields from 0.5 to 15.4 mg over 300 puffs; this represents approximately 21%-85% of the nicotine present in cartridges across products [11]. The chemistry and formulation of ENDS also play a role. Nicotine salt aerosols, nicotine combined with organic acids to form a less irritating compound, can reduce harshness and increase appeal. Furthermore, the relatively low odor and decrease in smoke compared to cigarettes may increase convenience and facilitate more frequent or discreet use. The heightened appeal and ease of use can lead to rapid increase in nicotine intake and faster development of dependence in some users [12,13].

When nicotine exposure abruptly stops or drops markedly, nicotine withdrawal results, reflecting both reduced nicotinic receptor stimulation and downstream dysregulation of dopamine, norepinephrine, serotonin, and acetylcholine pathways [14,15]. Withdrawal symptoms are generally similar between vaping and traditional cigarette use, though vaping may produce more variable onset and intensity due to differences in nicotine delivery and formulations [9,11,14]. Clinically, nicotine withdrawal presents as negative affect, anxiety, irritability or agitation, sleep disruption, and cognitive slowing [14,15]. Meta-analytic evidence in tobacco abstinence shows measurable increases in negative mood and anxiety symptoms even during early abstinence, supporting the possibility of ED-relevant symptom surges after nicotine withdrawal [16].

Adolescents appear uniquely vulnerable to the acute psychiatric effects of nicotine withdrawal following vaping cessation. Nicotine exposure during neurodevelopment can produce persistent alterations in neuronal signaling and behavior. This likely boosts susceptibility to mood or anxiety dysregulation and sustains cycles of withdrawal-relief use [17]. This developmental vulnerability may help explain why youth often report strong cravings and difficulty quitting when they attempt to stop vaping [4-6].

Clinical psychiatric manifestations relevant to emergency settings

Nicotine withdrawal often presents with anxiety and panic-spectrum symptoms such as intensified anxiety, inner restlessness, and psychomotor agitation. In some groups, symptom severity correlates more with nicotine dependence than with preexisting anxiety or depressive disorders [8]. In emergency departments, these symptoms may mimic panic disorder, appearing as acute panic attacks, pronounced agitation, inability to remain still, and a symptom profile combining insomnia and autonomic hyper arousal. These panic-like withdrawal presentations, timing, and distinguishing diagnostic cues are summarized in Table 1.

**Table 1:** Emergency-relevant psychiatric presentations after nicotine vaping cessation/withdrawal: timing, diagnostic cues, and ED management Abbreviations: ED = emergency department; ENDS = electronic nicotine delivery systems; NRT = nicotine replacement therapy; SI = suicidal ideation.

ED presentation cluster	Typical ED presentation	Usual withdrawal timing	Key diagnostic cues suggesting nicotine withdrawal	ED priorities & first-line actions	Key supporting references
Anxiety / panic-like hyperarousal	Panic attacks, dyspnea, chest tightness, tremor, pacing, intense internal restlessness	Onset hours–Day 1, peak often within first week, gradual resolution over 2–4 weeks	Clear temporal link to last vape; co-occurrence with irritability, insomnia, craving, concentration difficulty; partial symptom relief after nicotine stabilization	Rule out cardiopulmonary emergencies as indicated; obtain detailed vaping history (device, nicotine strength, last use); supportive de-escalation; consider nicotine replacement therapy (NRT) and reassess	[1-3,27,28]
Irritability / agitation / behavioral dyscontrol	Anger, verbal aggression, impulsivity, elopement attempts, escalating conflict	Early (Day 0–1), frequently intensifies during first week	Worsens during forced abstinence (boarding/hospitalization); improves when withdrawal physiology addressed; accompanied by insomnia and craving	Evaluate for delirium and medical precipitants; de-escalation strategies; avoid reflexive heavy sedation when withdrawal plausible; consider NRT when appropriate	[2,19-21,27,29]
Dysphoria / depressed mood / anhedonia	Tearfulness, low mood, anhedonia, hopelessness, fatigue	Commonly Day 1–Week 1, may persist variably	Rapid onset after cessation; negative affect linked more to dependence severity than baseline depression/anxiety liability	Suicide risk assessment; treat sleep disturbance and withdrawal contributors; consider NRT; avoid premature diagnosis of new major depressive episode	[3,8,16,30]
Insomnia with cognitive disruption	Severe insomnia, “brain fog,” impaired concentration, emotional lability	Often begins Day 0–1, drives symptom severity through first week	Sleep loss acts as a symptom amplifier; co-occurs with anxiety/agitation during withdrawal	Reduce environmental stimulation; sleep-supportive care; consider NRT to blunt withdrawal arousal; reassess mental status after sleep restoration	[1,2,21]
Suicidal ideation (SI) or self-harm risk amplification	Passive or active SI emerging after quitting; impulsive self-harm thoughts during severe dysphoria/insomnia	Variable; often overlaps with peak negative affect in early abstinence	Withdrawal can amplify suicidality but should never be considered the sole cause; e-cigarette use associated with suicidal behaviors in observational syntheses	Full suicide risk assessment; manage crisis regardless of presumed etiology; treat withdrawal contributors (sleep, nicotine stabilization); safety planning and close follow-up	[16,22,30,31]
Psychotic or perceptual symptoms (rare)	New hallucinations, paranoia, severe derealization, disorganized behavior	Rare; reported in case-level contexts with confounding factors	Must prioritize medical, toxicologic, and substance-induced causes; withdrawal considered only when tightly time-linked and improves with stabilization	Full medical/toxicology evaluation; manage agitation safely; consider nicotine withdrawal as contributing factor; reassess after stabilization before diagnosing primary psychosis	[21,23,24]
Populations at higher risk for severe withdrawal	Adolescents/young adults; high-nicotine pod or disposable users; heavy daily users; psychiatric comorbidity	N/A	Youth neurodevelopmental vulnerability; high nicotine delivery and dependence severity	Explicitly ask about device type, nicotine concentration (salt vs free-base), time-to-first-use, daily frequency, last use time	[1,4,5,9,12,17]
Rationale: modern ENDS and withdrawal intensity	High dependence and withdrawal burden in some vapers	N/A	Nicotine delivery from some ENDS approaches cigarettes; nicotine salt formulations increase acceptability and intake	Use delivery/dependence context to justify withdrawal diagnosis and management plan	[9,11-13]
Disposition and cessation bridge	Stabilized patient motivated to quit	N/A	Withdrawal recognized and treated improves safe discharge	Provide structured cessation follow-up; reference emerging pharmacotherapy evidence	[31-33]

Nicotine withdrawal leads to dysphoria, anhedonia, and irritability by disturbing reward processes during abstinence, which can drive impulsive and reactive behavior [8]. Studies show that irritability, anxiety, and restlessness are common in both current and former smokers, highlighting how frequently these symptoms occur during nicotine withdrawal [7,8,14].

In acute medical settings, nicotine withdrawal has been repeatedly implicated as a contributor to agitation and delirium-like syndromes particularly among hospitalized and intensive care populations. However, interpretation is limited by substantial confounding from critical illness severity, sedative exposure, and co-occurring alcohol withdrawal. Nevertheless, multiple ICU-based studies have demonstrated associations between abrupt nicotine abstinence and increased agitation or delirium. This supports a continuum of withdrawal-related neuropsychiatric risk that begins in the emergency department and extends into inpatient care [18-20]. Table 1 outlines key features that help distinguish nicotine withdrawal-associated agitation from delirium and other medical or substance-induced syndromes in emergency settings. 

During nicotine withdrawal, affective volatility may plausibly worsen suicidal ideation and self-harm risk in vulnerable individuals by intensifying dysphoria, irritability, and impulsivity. While systematic reviews and meta-analyses of observational studies report associations between e-cigarette use and suicidal ideation or behaviors, causal inference is limited by substantial confounding from underlying depression, poly-substance use, and psychosocial adversity [21]. For emergency psychiatry, the key clinical implication is pragmatic: nicotine withdrawal may amplify suicidal thinking even when not the primary cause and should therefore be routinely assessed and addressed as a modifiable contributor during acute crisis stabilization. Emergency-relevant considerations for assessing and managing suicidal ideation in the context of suspected nicotine withdrawal are summarized in Table 1. 

Psychotic or perceptual symptoms may emerge or worse during periods of nicotine exposure or withdrawal. A systematic review has synthesized evidence of associations between e-cigarette use and psychosis-related outcomes; however, the literature is heterogeneous and frequently confounded [22]. Additionally, case reports and series describe acute psychotic presentations temporally linked to vaping or withdrawal, including reports of nicotine e-liquid dependence with psychosis after cessation. These remain low-level evidence and should be interpreted cautiously [23].

The onset of nicotine withdrawal symptoms typically begins within hours of cessation, often emerging as early as two to 12 hours after the last nicotine exposure due to rapid nicotine clearance and loss of receptor stimulation. Early symptoms include anxiety, restlessness, irritability, and autonomic arousal, frequently accompanied by insomnia and difficulty concentrating. Symptom intensity peaks within the first 48-72 hours, with affective lability, agitation, dysphoria, and impulsivity most pronounced and often leading to emergency department presentation [24]. In the days and weeks that follow, somatic symptoms and autonomic hyper arousal gradually diminish. However, affective symptoms such as low mood, anhedonia, and irritability may persist, especially in those with higher nicotine dependence or psychiatric vulnerability. Most acute withdrawal symptoms resolve within two to four weeks, but residual mood instability and craving can fluctuate for months. This highlights the dynamic and evolving nature of withdrawal and its relevance to both acute crisis stabilization and short-term relapse risk [25].

High baseline dependence, psychiatric comorbidity, sleep deprivation, concurrent stimulant or cannabis use, and psychosocial stress can prolong or intensify symptoms, factors common among ED psychiatric presentations. Consequently, withdrawal is especially likely to amplify symptoms during acute crises [7,26].

Evidence from emergency and acute care settings

Direct emergency department studies that specifically isolate vaping cessation as the exposure remain limited. As a result, most clinically actionable evidence in acute care settings comes from two related bodies of literature. These include the broader nicotine withdrawal literature and hospital or ICU-based studies where abrupt nicotine deprivation can be clearly identified and temporally linked to agitation or delirium outcomes [18-20]. Pharmacologic and dependence studies of electronic nicotine delivery systems also demonstrate that many contemporary vaping products deliver nicotine doses sufficient to produce clinically significant withdrawal when use is abruptly discontinued [9,11,12].

**Figure 1:**
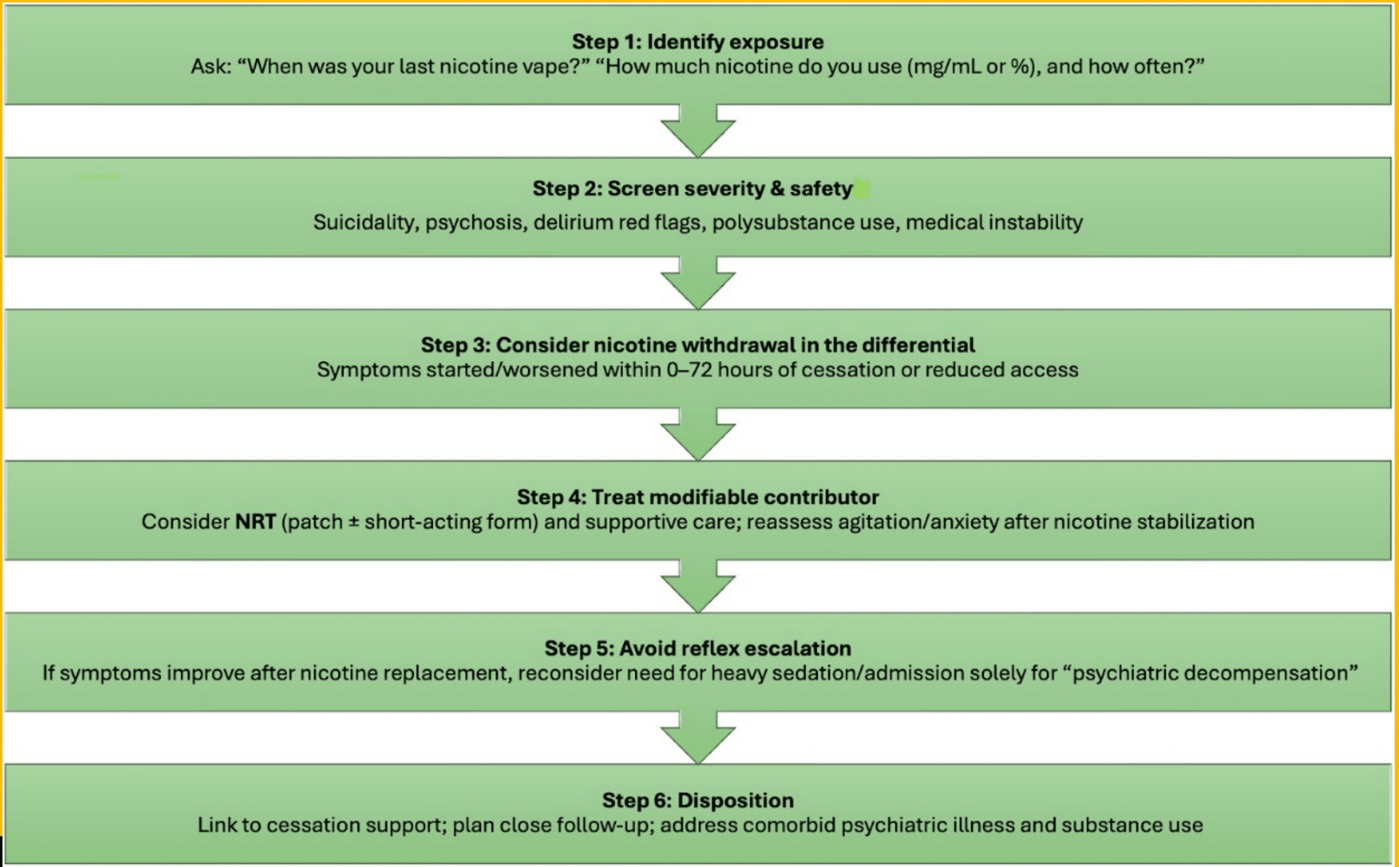
Practical ED approach (flow) to suspected nicotine vaping withdrawal presenting as acute psychiatric symptoms This figure was created by the authors for this manuscript

Emergency clinicians often see patients after sudden behavioral changes such as abrupt vaping cessation. Nicotine withdrawal is common, can be severe in dependent users, may mimic primary psychiatric illness, and can worsen agitation or suicidal risk in emergency presentations [16,24]. While ED-based studies of vaping cessation are needed, existing evidence supports routinely assessing for recent nicotine discontinuation as a modifiable contributor to acute psychiatric symptoms. A practical, stepwise ED approach to identifying and managing suspected nicotine vaping withdrawal presenting as acute psychiatric symptoms is outlined in Figure 1. 

Differential diagnosis and diagnostic pitfalls

In emergency psychiatry, a central diagnostic pitfall is misattributing nicotine withdrawal-driven symptoms to a primary psychiatric disorder. This misattribution can lead to escalation of antipsychotics or benzodiazepines, or involuntary hospitalization, often without addressing nicotine deprivation as a precipitating factor. Diagnostic confusion can be further compounded by possible dual diagnoses, as patients with mood or anxiety disorders may use nicotine as a form of self-medication, masking withdrawal-related symptom exacerbation [7,8]. To avoid this, a withdrawal-aware differential diagnosis requires careful attention to temporal relationships, particularly the onset of symptoms following abrupt cessation, and recognition of characteristic features such as prominent irritability, craving, insomnia, and autonomic arousal. These can help distinguish nicotine withdrawal from primary anxiety or mood disorders. Furthermore, substance-induced syndromes related to alcohol, cannabis, or stimulant use should be considered, as poly-substance exposure is common and may substantially confound clinical presentations. In medically ill or hospitalized patients, delirium and other medical mimics warrant careful evaluation, with nicotine withdrawal viewed as a potential contributor among multiple interacting precipitants rather than as an isolated cause [20]. Acute withdrawal phenomena may also resemble emerging psychotic disorders; however, existing observational data show association rather than causation, so these findings should be interpreted cautiously [22,23]. Ultimately, obtaining a brief but focused nicotine use history, including device type, nicotine concentration, frequency of use, time since last exposure, and prior withdrawal severity, is often the most informative step for clarifying diagnosis and guiding management.

Management considerations in emergency settings

Assessment strategies include nicotine vaping in the standard substance history, as many patients do not disclose vaping as “nicotine” or perceive it as substance use. This highlights a communication gap clinicians must recognize and address when obtaining a standard substance history. Dependence measures in youth studies show that symptoms like craving and failed quit attempts are prevalent and clinically significant [4-6].

Nicotine replacement therapy (NRT) is a symptom treatment. Although most high-quality data are from smoking cessation, NRT reliably reduces withdrawal symptoms and increases quit success; combination strategies can be more effective than single-form NRT [27-29]. In acute care, NRT has been studied primarily for safety/feasibility, especially in ICU contexts where withdrawal can complicate agitation and sedation plans [20].

Avoid unnecessary pharmacologic escalation or hospitalization. Managing nicotine withdrawal does not substitute for standard emergency psychiatry (safety, medical evaluation, primary illness management), but it reduces symptom overlap and prevents excessive treatment. The EAGLES trial confirms that standard smoking-cessation medications (varenicline, bupropion, nicotine patch) did not show significant neuropsychiatric harm among smokers with or without psychiatric illness [30].

Vaping cessation interventions (2010-2025 evidence). Evidence for vaping-specific cessation interventions is developing. A 2025 Cochrane review compiles randomized trials of vaping cessation interventions [31]. Pharmacotherapy trials in adults signal promise for cytisinicline and varenicline in vaping cessation, but these require follow-up infrastructure, not immediate ED use [32,33]. ED clinicians can start the bridge: identify withdrawal, manage symptoms, and connect patients to ongoing cessation support.

Special populations

Certain groups are especially vulnerable to psychiatric effects during nicotine vaping withdrawal. Adolescents and young adults are at high risk due to frequent vaping and heightened fronto-limbic sensitivity, which likely intensifies mood and anxiety instability during withdrawal [1,2,17]. Those with existing psychiatric disorders, especially anxiety or depression, may have exacerbated symptoms during cessation, as high anxiety sensitivity and low distress tolerance correlate with greater dependence, more withdrawal, and worse outcomes [7]. Hospitalized and medically ill patients are also at risk, since abrupt nicotine deprivation in inpatient and ICU settings is linked to agitation and delirium-like syndromes, complicating sedation and behavior management [18-20]. Dual users and those using high-delivery devices may receive high, variable nicotine doses; studies show significant nicotine exposure and craving patterns that can worsen withdrawal if access is suddenly cut off [34].

Limitations of current evidence

The main limitation in current literature is the lack of emergency department-specific studies that evaluate psychiatric outcomes after vaping cessation. Consequently, most evidence extrapolates from general nicotine withdrawal data and from hospital or ICU studies where nicotine deprivation is more identifiable. This may limit generalizability to emergency psychiatric settings and highlights the need for future, ED-focused studies. Reported links between e-cigarette use and outcomes like suicidality or psychosis come mainly from observational data and remain speculative [21,22]. While case reports describe acute neuropsychiatric symptoms linked to vaping cessation, these are limited by selection bias and lack broader applicability [23].

Clinical implications

For emergency psychiatry, the key message is clear: if you don’t ask about vaping, you will overlook a common, treatable factor in acute psychiatric presentations. Identifying nicotine withdrawal can: improve diagnostic accuracy [1]; reduce unnecessary sedation or escalation [2]; improve patient experience [3]; and potentially lower safety risks when withdrawal fuels suicidality or agitation [4].

Future research directions

Critical next steps include prospective ED studies tracking psychiatric symptoms after vaping cessation using clear measures of nicotine dependence severity, time since last use, biomarkers (e.g., cotinine), and standardized scales. Pragmatic ED trials should assess whether early nicotine withdrawal treatment, especially with NRT, reduces agitation, suicidal ideation, and unnecessary escalation. Future work must also distinguish vaping cessation effects from co-existing psychiatric illness or poly-substance use, which often complicate acute cases. Ongoing randomized trials of vaping cessation guide ED-initiated care models that link stabilization with continued treatment [31-33].

## Conclusions

Nicotine vaping cessation can precipitate acute psychiatric symptoms, especially anxiety, irritability/agitation, dysphoria, insomnia, and cognitive disruption that commonly resemble primary psychiatric illness. Overall, the withdrawal symptoms of ENDS closely parallel those of traditional cigarette cessation; however, higher nicotine delivery, more frequent dosing, and user perception that vaping is less harmful may intensify symptoms in some individuals. In acute-care contexts, nicotine withdrawal may also contribute to agitation/delirium phenotypes and complicate risk assessment. Emergency clinicians should treat vaping history as essential, incorporate withdrawal into the differential diagnosis, and consider NRT-based symptom stabilization with clear follow-up planning.
